# Reply to B. Meunier’s Letter to the Editor Re: Brewer G. J.; *Nutrients* 2015, *7*, 10053–10064

**DOI:** 10.3390/nu8080517

**Published:** 2016-08-22

**Authors:** George J. Brewer

**Affiliations:** Human Genetics, University of Michigan, 3820 Gensley Road, Ann Arbor, MI 48103, USA; brewergj@umich.edu; Tel.: +1-734-761-7970

In a letter to the editor, Meunier [1] apparently attempts to discredit the copper-2 hypothesis for causation of the Alzheimer’s disease (AD) epidemic in developed countries proposed by myself in a review in this journal [2]. Briefly, the review laid out the following scenario: First, that AD was rare, perhaps at about a 1% prevalence level, in developed countries prior to 1900. The evidence for this is that esteemed clinicians of the period, Osler [3], Gower [4], and Freud [5] did not report any patients with an AD-like disease, and pathologists of the period, such as Boyd [6], did not report brains at autopsy with amyloid plaques and neurofibrillary tangles, hallmarks of AD brain pathology. Second, developed countries have undergone a huge increase in AD prevalence during the 20th century, an “AD epidemic”. Third, that undeveloped countries have not shared in the AD epidemic, remaining, at about 1% prevalence. All of this was well documented by very solid referencing [2]. Fourth, that the huge difference in AD prevalence in developed countries between the 19th and 20th centuries was not due to a lack of old people in developed countries during the 19th century, which is relevant because aging is a major risk factor for AD. Fifth, that the above four facts can only be explained by an environmental cause in developed countries in the 20th century. In other words a new environmental agent or agents had to have emerged in the 20th century in developed countries to cause the AD epidemic. Sixth that increased divalent copper, or copper-2, ingestion was at least part of that environmental causal change. Lack of space here precludes reiterating in detail all the evidence for copper-2 causality which was carefully laid out in my review. In brief, copper-2 ingestion from drinking water in AD animal models greatly enhanced the AD-like disease [7] and copper-2 ingestion in copper supplement pills in humans caused rapid loss of cognition [8]. It was shown that the AD epidemic in developed countries closely paralleled time-wise the huge increase in use of copper plumbing [2] and that enough copper was leached from copper plumbing to be causal of AD using the animal models as a guide [2].

A key factor in the structure of the hypothesis of copper-2 causation of the AD epidemic in developed countries is that there were enough old people in developed countries in the 19th century to exhibit numerous cases of AD at today’s rate, so an increased aging population isn’t a simple explanation for the AD epidemic, since age is a major risk factor for AD.

There were two pieces of evidence cited to support the assertion that there were enough old people in developed countries in the 19th century to have AD patients show up in clinics and provide brains at autopsy with plaques and tangles. One was a reference to Waldman and Lamb [9] who stated that in 1911 half the population of France lived to age 60, and at today’s prevalence rate, at least 10% of them would have AD. The second piece of evidence was that in 1900, the US census showed that there were 3.6 million people age 60 or older, which at today’s prevalence rate would have generated 360,000 cases [2].

Meunier [1], first of all, incorrectly attempts to discredit my review by saying that I don’t believe age is a risk factor for AD. He even titles his paper “Age and Alzheimer’s Disease”. In his paper he states, “to discard the hypotheses of AD as being an aging disease, a commonly accepted idea, the author mentioned that half of the population of France lived to 60 or older in 1911 with a low prevalence of sporadic AD”. Far from “discarding the hypothesis of AD being an aging disease” I stated explicitly in my review [2], in Section 3, Risk Factors for AD “Age is a major risk factor” and again in Section 9, “and aging of course, is a very important risk factor”. It is inappropriate of Meunier [1] to state that I have discarded “the hypothesis of AD as being an aging disease” when the review clearly states the opposite.

A major point Meunier [1] tries to make is that I am wrong about the French data. He states that age 60 was the “median age” for the French population in 1911, and that only 12.7% of the French population was over age 60 in 1911. Meunier fails to realize that it isn’t the percentage of elderly people but their absolute number that would be critical in evaluating whether AD patients would be seen in clinics and the autopsy table at a noticeable frequency. Picking up on that theme and elaborating further on the data provided by Waldman and Lamb [9], they show an age pyramid for the 1911 population of France, on p 71 of their book. The pyramid shows there were about 255,000 people aged 65 to 75, which at the prevalence rate of 15% published in the current Alzheimer’s Disease Fact and Figures [10] would generate 52,500 patients. The pyramid shows about 115,000 people aged 75 to 85, which at the published prevalence rare of 44% would generate 50,000 cases. Thus, there would have been well over 100,000 AD patients in France in 1911, which would have provided plenty of patients to be seen in clinics and at the autopsy table. This gives a very different perspective than the 12.7% of the French population over age 60 quoted by Meunier [1].

Besides misinterpreting the French data, Meunier [1] ignores a second and more major piece of data supporting my contention that there were enough older people around in the 1900s to have shown up in clinics and at autopsy. There data are in the 1900 US census, which showed 3.6 million people age 60 or older in the US in 1900 [2]. At today’s prevalence rate, 360,000 AD patients would have resulted, more than enough to show up in clinics and at the autopsy table.

Meunier [1] not only does not address the US data discussed above, but he ignores the huge difference in current AD prevalence in developed vs. undeveloped countries in people of the same older ages which I carefully document with good references in the review [2]. This difference, as well as the difference in AD prevalence in the 19th vs. the 20th centuries in developed countries, supports my conclusion in the review that an environmental agent or agents arising in developed countries in the 20th century is responsible for the AD epidemic. As discussed earlier, a good case is made in the review that those agents are copper-2 from drinking water and copper supplement pills, and increased meat eating, which increases copper absorption [2]. Meunier [1] does not discuss any of these data, focusing narrowly on what he mistakenly thinks is my misinterpretation of the number of older people in France in 1911, thus unfairly trying to give a negative impression of the entire review.

The conclusion that new environmental factors are responsible for the AD epidemic in developed countries is important, because it provides impetus to find them, mitigate them, and thus save tens of millions of people from losing their “golden years” due to this disastrous disease.
